# NFYB Integrates Hormonal Signals into Tissue Allometry by Promoting Protein Biosynthesis

**DOI:** 10.1002/advs.75767

**Published:** 2026-05-20

**Authors:** Fangfang Liu, Shiming Zhu, Sishi Xia, Xiaoshuai Zhang, Qin Li, Zhanquan Zeng, Yuqing Liu, Wanyin Liang, Guanyu Zhou, Erxia Du, Suning Liu, Sheng Li

**Affiliations:** ^1^ Guangdong Provincial Key Laboratory of Insect Developmental Biology and Applied Technology Institute of Insect Science and Technology School of Life Sciences South China Normal University Guangzhou China

**Keywords:** 20‐hydroxyecdysone, fat body, juvenile hormone, protein biosynthesis, wing pads

## Abstract

Tissue allometry is under precise hormonal control for achieving the appropriate body size and shape. While juvenile hormone (JH) and 20‐hydroxyecdysone (20E) coordinate insect growth and development, their roles in regulating tissue allometry remain unclear. During molting cycles in the American cockroach, we reveal that JH promotes early, drastic fat body expansion, whereas 20E stimulates late, rapid wing pad growth. Through transcriptomic screening of early and late fat body and wing pads across the last five nymphal instars, plus JH‐treated fat body and 20E‐treated wing pads, we identify *NFYB* as the central spatiotemporal factor gene. Mechanistically, by transcriptionally activating the core translational machinery, NFYB promotes protein biosynthesis of hexamerin storage compounds in the early fat body and minichromosome maintenance complex in the late wing pads. Our findings demonstrate NFYB as a spatiotemporin that integrates different hormonal signals into distinct tissue allometry, shedding light on how appropriate body shape is achieved in animals.

## Graphical abstract

In the American cockroach, NFYB acts as a spatiotemporin that translates distinct hormonal cues into tissue‐specific allometry. Juvenile hormone activates NFYB in the early fat body, while 20‐hydroxyecdysone induces it in late wing pads. NFYB then promotes protein biosynthesis via core translational machinery, driving differential growth across the molting cycle.

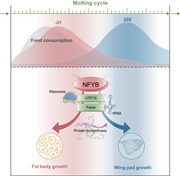

## 1 Introduction

Tissue allometry, which refers to the differential scaling of body parts with body size or of various body parts, is a fundamental trait in animals that enables efficient coordination of body proportions for adapting to diverse ecological and physiological demands [1, 2, 3, 4, 5]. For instance, carnivores with large heads capable of capturing sizable prey exhibit reduced neck proportions, while herbivores develop relatively small heads and long necks to expand their feeding range [6]. In humans and mammals, some neonatal organs (e.g., brain and liver) undergo rapid growth to meet high metabolic and cognitive demands, which are controlled by the growth hormone [7, 8]; conversely, reproductive organs undergo accelerated development prior to sexual maturity, which are predominantly regulated by sexual steroidal hormones [9, 10, 11]. Pathologically, congenital birth defects such as phocomelia (malformation of the limbs) and achondroplasia (disproportionate dwarfism) arise from allometric growth [12]. While hormones are known to play essential roles in regulating tissue allometry [1, 2, 13, 14, 15], we hypothesize that a dedicated transcriptional integrator exists to integrate diverse hormonal signals into distinct tissue allometry, thereby orchestrating body size and shape during animal ontogeny. We refer to such a hypothesized factor as a “spatiotemporin”.

Insects undergo multiple larval or nymphal molts to reach their final body size. Two major insect hormones, juvenile hormone (JH) and 20‐hydroxyecdysone (20E), coordinate growth, development, molting, and metamorphosis [16, 17]. Mechanistically, JH binds to its intracellular receptor complex Met/Tai and subsequently induces the expression of the anti‐metamorphic factor *Kr‐h1*, which represses the 20E‐EcR/USP‐triggered transcriptional cascade (including the pupal specifier *Broad* and the adult specifier *E93*) to maintain the larval or nymphal status [18, 19, 20, 21, 22, 23, 24, 25, 26, 27, 28]. By contrast, 20E signaling not only ensures the periodic larval or nymphal molts, but also induces *Broad* expression to trigger pupation and *E93* expression to trigger adult formation, respectively [16, 29]. Experimentally, exogenous JH treatment or *E93* knockdown can induce supernumerary molts, leading to larger body size [27], whereas knockdown of *Met* or *Kr‐h1* and overexpression of *Broad* can cause precocious metamorphosis with reduced body size [22, 30, 31, 32]. Moreover, JH and 20E are also involved in regulating tissue allometry. For example, JH affects a number of exaggerated phenotypic traits, including enlarged eyestalks of stalk‐eyed flies [33], mandibles of stag beetles [34], flour beetles [35], and termite soldiers [36], and horns of dung beetles [37, 38]. Likewise, 20E promotes allometric growth of the wing disc at certain developmental stages in *Drosophila* [39, 40, 41] and Lepidoptera [42, 43, 44]. Moreover, tissue allometry involves complex interplays between JH and 20E [12]. Nevertheless, nothing is known about whether JH and 20E can act through a hypothetical spatiotemporin to regulate tissue allometry for controlling insect body size and shape.

Most insights into insect allometry are derived from holometabolous models, such as *Drosophila* and *Manduca sexta*, which have a few larval instars and a fast growth duration [42, 45, 46, 47]. To achieve precise spatiotemporal resolution of the dynamics of developmental growth, we turned to the hemimetabolous American cockroach, *Periplaneta americana*. This species possesses a suite of distinctive advantages in studying tissue allometry, including large body size, slow growth rate, and up to 14 nymphal molting cycles [48]. These strengths enable precisely segmenting each molting cycle into discrete early, mid, and late stages. This temporal segmentation, coupled with the high RNAi efficiency reported in this species [49, 50, 51, 52, 53], establishes it as an exceptional system for investigating the spatiotemporal control of tissue allometry during the nymphal molting cycles. Interestingly, we observed that food intake, whole body growth, and fat body expansion simultaneously occur at the early stages of the molting cycles, while wing pads grow fast at the late stages. We thus observed positive allometry in early fat body and late wing pads, and revealed that JH and 20E promote the allometry of fat body and wing pads, respectively. Importantly, we identified a nuclear transcription factor Y subunit beta (NFYB) as the spatiotemporin that integrates JH and 20E signals into distinct tissue allometry by promoting stage‐ and tissue‐specific protein biosynthesis. Thus, this study not only supports our spatiotemporin hypothesis in controlling body size and shape in insects, but also provides a framework to further identify similar key factors within and across species in animals.

## 2 Results

### 2.1 JH Promotes Early Fat Body Expansion and 20E Stimulates Late Wing Pad Growth

**FIGURE 1:**
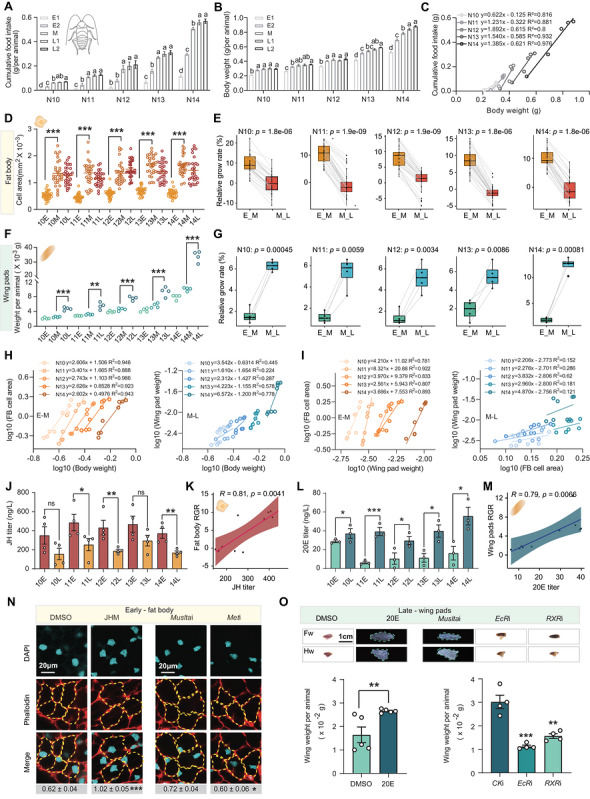
JH and 20E promote fat body expansion and wing pad growth, respectively, during the final five nymphal instars. (A) Cumulative food intake during five developmental substages (E1, E2, M, L1, L2) per molting cycle (10th – 14th instars). (B) Body weight across five developmental substages (E1, E2, M, L1, L2) per molting cycle (10th – 14th instars). (C) Correlation between cumulative food intake and body weight during the final five nymphal instars. (D) Fat body cell area across three developmental substages (E, M, L) per molting cycle (10th – 14th instars). (E) Relative growth rate of fat body cells (10th – 14th instars). (F) Wing pad weight across three developmental substages (E, M, L) per molting cycle (10th – 14th instars). (G) Relative growth rate of wing pads (10th – 14th instars). (H) Allometric analysis of fat body versus whole‐body growth during the early‐to‐mid nymphal stages (left), and wing pad versus whole‐body growth during the mid‐to‐late nymphal stages (right). (I) Allometric analysis of fat body versus wing pad growth during the early‐to‐mid nymphal stages (left), and wing pad versus fat body growth during the mid‐to‐late nymphal stages (right). (J) JH titer during early and late substages per molting cycle (10th – 14th instars). (K) Correlation of JH titer with fat body growth. (L) 20E titer during early and late substages per molting cycle (10th – 14th instars). (M) Correlation of 20E titer with wing pad growth. (N) Fat body cell morphology after treatment with methoprene (JH mimic, JHM) or *Met* RNAi. (O) Wing pad morphology and weight after treatment with 20E and *EcR/RXR* RNAi. E: early, M: middle, L: late. Scale bars: 20 µm (N), 1 cm (O, left). Data are shown as mean ± SEM; the same method was employed for all data analyses. Different letters above the bars indicate statistically significant differences. (*p* < 0.05). ^*^ *p* < 0.05, ^**^ *p* < 0.01, ^***^ *p* < 0.001, compared to the negative controls.

During our initial observations, we noticed that cockroach nymphs fed voraciously during the early stage of a molting cycle but consumed very little at the late stage. To quantify the feeding patterns across the final five nymphal instars, we found that feeding predominantly occurred during the early‐mid stages but dramatically declined at the mid‐late stages (Figure 1A). Correspondingly, the developmental pattern of body growth closely mirrored that of food intake across the molting cycles (Figure 1B), and correlation analysis further confirmed their robust positive relationship during each molting cycle (R^2^ = 0.8–0.976) (Figure 1C). Having established this intriguing organism‐level phenomenon, we next addressed how this systemic growth is coordinated by tissue allometry. Comparative histomorphometric analyses revealed that the fat body underwent drastic expansion during the early‐mid feeding stages, while it maintained a stable size or slightly declined at the mid‐late non‐feeding stages (Figure 1D,E) [54]. In contrast, wing pads exhibited rapid growth during the mid‐late stages but grew very little at the early‐mid stages (Figure 1F,G). Further analysis during the last molting cycle showed that fat body expansion followed logistic growth primarily during the early‐mid stages, whereas wing pad growth exhibited exponential growth parameters predominantly at the mid‐late stages (Figure S1C–H).

In 1936, Tessier and Huxley proposed the law of simple allometry, which can be expressed as the formula y = bx^α^, where α is the ratio of growth rate that is called the allometric coefficient (α = 1, isometry; α > 1, positive allometry; α < 1, negative allometry) between trait x and trait y [2]. When the equation is log transformed, a linear relationship log(y) = log(b) + α log(x) can be obtained [12]. We then examined the potential allometric growth of fat body and wing pads across the final five nymphal instars. Notably, fat body cell area scaled with positive allometry relative to body weight during the early‐mid stages (α = 2.602–3.401), but switched to negative allometry at the mid‐late stages (α = 0.337–0.788) (Figure 1H; Figure S1I). Conversely, wing pad weight exhibited negative allometry relative to body weight during the early‐mid stages (α = 0.401–0.893), but shifted to positive allometry at the mid‐late stages (α = 1.61–6.572) (Figure 1H; Figure S1J). Correspondingly, fat body expansion exhibited positive allometry relative to wing pad growth during the early‐mid stages (α = 2.561–8.321); in contrast, wing pad growth showed positive allometry relative to fat body growth at the mid‐late stages (α = 2.206–4.87) (Figure 1I). Given that food intake mainly promotes individual growth [55], we infer that nutritional signals first trigger drastic fat body expansion to accumulate biomass during the early‐mid stage of each molting cycle, while rapid wing pad growth is secondarily fueled at the following mid‐late stage (Figure S1K).

To examine the potential roles of insect hormones in regulating tissue allometry, we measured JH titers using quantitative liquid chromatography‐tandem mass spectrometry (LC‐MS/MS) and 20E titers using enzyme‐linked immunosorbent assay (ELISA) at the early and late stages across the final five molting cycles. The results showed that JH titers were always higher at the early stages than those at the late stages, and correlation analysis revealed a positive relationship between JH titers and the fat body expansion rates (R^2^ = 0.81) (Figures 1J,K). Conversely, 20E titers were always higher at the late stages than those at the early stages, with a positive relationship between 20E titers and the wing pad growth rates (R^2^ = 0.79) (Figure 1L,M). We thus assume that JH might promote early fat body expansion and 20E might stimulate late wing pad growth.

To examine the cause‐effect relationship, we functionally validated the potential correlations between JH and fat body as well as between 20E and wing pads through pharmacological and genetic approaches at the last molting cycle (Figure S2A). Exogenous application of the JH mimic methoprene (JHM) promoted fat body cell expansion both in vivo (Figure 1N; Figure S2C) and in vitro (Figure S2B, B’), while RNAi knockdown of the JH intracellular receptor *Met* suppressed it (Figure 1N; Figure S2C). Likewise, direct 20E injection stimulated wing pad growth (Figure 1O; Figure S2D), and RNAi knockdown of the 20E nuclear receptor *EcR* and *RXR* inhibited it (Figure 1O; Figure S2D). To further validate the role of 20E, we performed a neck ligation experiment (Figure S2E), which dramatically decreased 20E titer (Figure S2F) and suppressed wing pad growth (Figure S2G, G’). Importantly, the inhibition of wing pad growth by neck ligation could be rescued by exogenous 20E treatment (Figure S2G, G’). In summary, these results demonstrate that JH promotes early fat body expansion and 20E stimulates late wing pad growth during each molting cycle, thus orchestrating distinct tissue allometry that matches the periodic molting cycles.

### 2.2 NFYB Periodically Translates JH and 20E Signals Into Distinct Tissue Allometry

**FIGURE 2:**
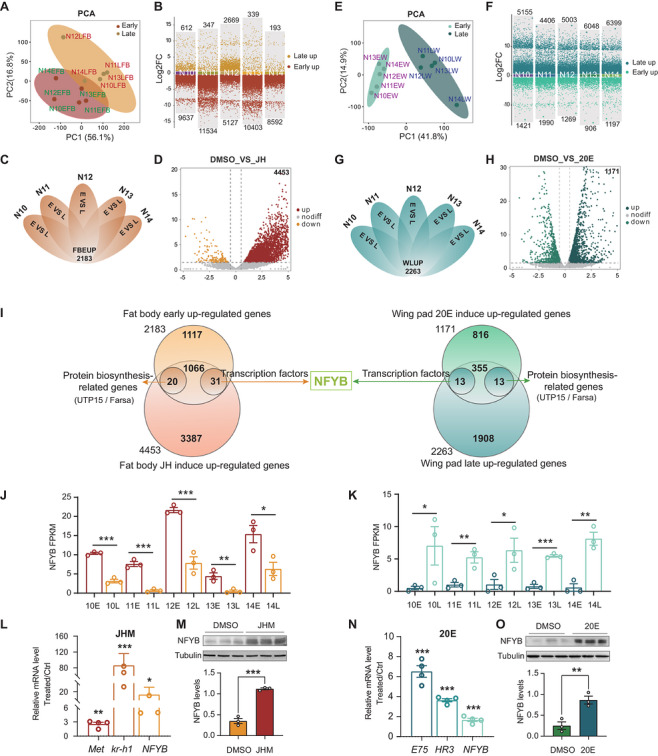
Hormone responsive NFYB functions as a stage‐specific transcription factor coordinating early fat body expansion and late wing pad growth. (A) Principal component analysis (PCA) of fat body samples grouped by early (E) and late (L) developmental stage across molting cycles (10th – 14th instars). (B) Stage‐specific differentially expressed genes (DEGs) in the fat body between E and L stages per molting cycle (10th – 14th instars). (C) Venn diagrams identifying upregulated genes in the fat body during E stages. (D) Volcano plots of DEGs in the fat body after treatment with JHM. DMSO serves as a control. (E) Principal component analysis (PCA) of wing pad samples grouped by E and L developmental stage across molting cycles (10th – 14th instars). (F) Stage‐specific DEGs in the wing pads between E and L stages per molting cycle (10th – 14th instars). (G) Venn diagrams identifying upregulated genes in the wing pads during E stages. (H) Volcano plots of DEGs in the wing pads after treatment with 20E. DMSO serves as a control. (I) Venn diagrams identifying overlapping genes between: early‐stage upregulated and JHM‐induced genes in the fat body (left); late‐stage upregulated and 20E‐induced genes in the wing pads (right). (J and K) The expression of *NFYB* in the fat body (J) and wing pad (K) during E and L substages across the molting cycle (10th – 14th instars). (L) The expression of *Met*, *Kr‐h1*, and *NFYB* after JHM treatment in the fat body. (M) Western blot analysis (upper) and quantification (lower) of NFYB protein levels after JHM treatment in the fat body. (N) The expression of *E75*, *HR3*, and *NFYB* after 20E treatment in the wing pads. (O) Western blot analysis (upper) and quantification (lower) of NFYB protein levels after 20E treatment in the wing pads. ^*^ *p* < 0.05, ^**^ *p* < 0.01, ^***^ *p* < 0.001, compared to the negative controls.

To examine our hypothesis that a potential spatiotemporin might periodically translate diverse hormone signals into distinct tissue allometry, we first compared the transcriptomic profiles of fat body and wing pads across early and late stages during the final five nymphal instars. Principal component analysis (PCA) segregated samples by developmental stage rather than instar, with early‐stage samples clustering separately from late‐stage samples in each tissue (Figure 2A,E). Differential expression analysis showed that gene upregulation mainly occurred at early fat body and late wing pads, corresponding to their fast‐growing phases (Figure 2B,F). Venn analysis identified 2183 and 2263 genes commonly upregulated in early fat body and late wing pads, respectively (Figure 2C,G). Meanwhile, following JH and 20E treatments at the last nymphal instar, we performed transcriptome profiles on the middle stage of the fat body and the late stage of wing pads, respectively. The results showed that JH upregulated 4453 genes in the fat body (Figure 2D), while 20E upregulated 1171 genes in the wing pads (Figure 2H). Hence, we analyzed the overlapping upregulated genes between the stage‐specific (Figure 2C,G) and the hormone‐induced transcriptomes (Figure 2D, H). The results revealed that 1066 genes were co‐upregulated in the fat body and 355 genes were co‐upregulated in the wing pads (Figure 2I).

Given the pivotal role of transcription factors (TFs) in orchestrating gene expression and developmental processes [56, 57], we focused on the TFs within these overlapping gene sets, revealing 31 TFs in the fat body and 13 TFs in the wing pads (Figure 2I; Figure S3A,B). Pronouncedly, we found that *NFYB*, encoding a subunit of the conserved CCAAT‐binding nuclear transcription factor Y (NF‐Y) complex [58, 59, 60], was the uniquely common TF gene identified in both tissues (Figure 2I). Extracted from the transcriptome data, we found that *NFYB* was upregulated at early fat body and late wing pads (Figure 2J,K). Furthermore, treatment of the fat body with JHM or wing pads with 20E increased *NFYB* expression at both mRNA and protein levels (Figure 2L–O). By contrast, *NFYB* expression was inhibited by RNAi knockdown of the JH nuclear receptor *Met* in the fat body or the 20E nuclear receptor *EcR* in the wing pads (Figure S3C,D). Overall, these results strongly suggest that NFYB might translate JH and 20E signals into allometric growth of early fat body and late wing pads during the nymphal molting cycles, respectively.

Meanwhile, through analysis of stage‐specific upregulated and hormone‐induced gene sets, we discovered two valuable cues: (1) KEGG analysis enriched several protein biosynthesis‐related pathways in both the fat body and wing pads (Figure S4A–H). Functional validation showed that JHM treatments elevated protein levels and stimulated *de novo* protein biosynthesis in the fat body (Figure S4I‐K’), and the stimulatory effects were able to be abolished by *Met* knockdown. Comparable experiments verified 20E‐dependent protein biosynthesis in the wing pads (Figure S4L–N’). (2) Protein biosynthesis‐related genes were enriched in both overlapping gene sets (20 genes in the fat body and 13 genes in the wing pads) (Figure 2I; Figure S5A,B). Strikingly, two common protein biosynthesis‐related genes, *UTP15*, implicated in ribosome biogenesis, and *FARSA*, implicated in tRNA aminoacylation [61, 62, 63], were shared in both tissues (Figure 2I). Based on these results, we hypothesize that JH and 20E induce the expression of spatiotemporin *NFYB* at early fat body and late wing pads during the molting cycles, respectively, thereby promoting protein biosynthesis to orchestrate distinct tissue allometry.

### 2.3 NFYB Orchestrates Tissue Allometry by Promoting Protein Biosynthesis

**FIGURE 3:**
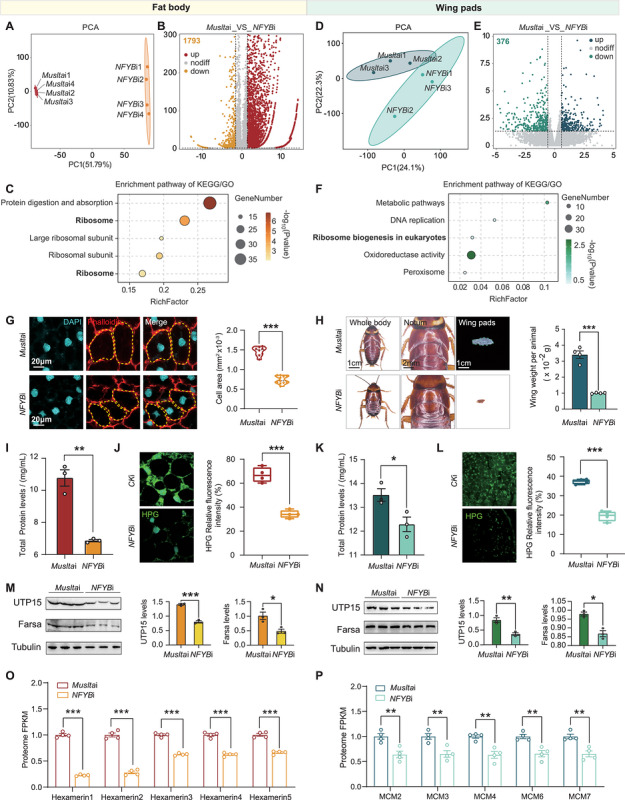
NFYB functions as a stage‐specific TF coordinating early fat body expansion and late wing pad growth by promoting protein biosynthesis. (A) PCA of fat body samples grouped by knockdown of *NFYB* and control. (B) Volcano plots of DEGs in the fat body between *NFYB* knockdown and control groups. (C) KEGG and GO analysis of fat body based on 1793 downregulated genes after *NFYB* knockdown. (D) PCA of wing pad samples grouped by knockdown of *NFYB* and control. (E) Volcano plots of DEGs in the wing pads between *NFYB* knockdown and control groups. (F) KEGG and GO analysis of wing pads based on 376 downregulated genes after *NFYB* knockdown. (G) Fat body cell morphology and quantification following *NFYB* knockdown. (H) Wing pad morphology and weight quantification following *NFYB* knockdown. Whole body (left), notum (middle), and wing pads (right) views. (I) Total protein levels in the fat body after *NFYB* knockdown. (J) Protein biosynthesis (HPG staining) in the fat body following *NFYB* knockdown. (K) Total protein levels in the wing pads after *NFYB* knockdown. (L) Protein synthesis (HPG staining) in the wing pads following *NFYB* knockdown. (M) Western blot analysis revealed reduced protein levels of UTP15 and Farsa following *NFYB* knockdown in the fat body. (N) Western blot analysis revealed reduced protein levels of UTP15 and Farsa following *NFYB* knockdown in the wing pads. (O) Levels of hemocyanin‐domain proteins following *NFYB* knockdown in the fat body. (P) Levels of MCM‐complex proteins following *NFYB* knockdown in the wing pads. Scale bars: 20 µm (G), 1 cm (H, left and right), 2 mm (H, middle). ^*^ *p* < 0.05, ^**^ *p* < 0.01, ^***^ *p* < 0.001, compared to the negative controls.

To verify the above hypothesis, we performed additional transcriptomic analyses following *NFYB* knockdown in both the fat body and wing pads. As expected, *NFYB* knockdown significantly reduced its transcript and protein levels in both tissues (Figure S6A, B). PCA revealed clear separation between control and *NFYB*‐depleted groups in both tissues (Figure 3A,D). Differential expression analysis identified 1793 and 376 genes significantly downregulated in the fat body and wing pads, respectively (Figure 3B,E). Functional enrichment analysis demonstrated that these downregulated genes are strongly associated with ribosome biogenesis pathways, which control protein biosynthesis (Figure 3C,F), consistent with the stage‐specific and hormone‐treated transcriptome (Figure 2A–I). Importantly, RNAi knockdown of *NFYB* reduced fat body expansion (Figure 3G) and wing pad growth (Figure 3H; Figure S6C), and resulted in a significant decline of protein biosynthesis (Figure 3I–L; Figure S6D, E). The results confirm the potential roles of NFYB in upregulating protein biosynthesis‐related genes (Figure 2I). Coincidentally, the common factors UTP15 and FARSA were significantly suppressed at both mRNA and protein levels upon *NFYB* knockdown (Figure 3M, N; Figure S6D, E). Notably, quantitative allometric analysis revealed that *NFYB* knockdown shifted the growth trajectory of the fat body from positive allometry (α = 2.623) to negative allometry (α = 0.341) relative to body weight, and reduced wing pad growth from positive allometry (α = 2.774) to near‐isometry (α = 1.138) (Figure S6F).

Finally, we conducted LC‐MS/MS proteomic analysis to investigate how *NFYB* knockdown terminally affected protein biosynthesis (Figure S7A). We identified 304 and 81 proteins significantly downregulated in the *NFYB*‐depleted fat body and wing pads, respectively (Figure S7B, C). Domain enrichment revealed that hexamerin storage compounds were prominently suppressed at the early stage of fat body during the last nymphal instar, whereas minichromosome maintenance (MCM) complex proteins were notably reduced at the late stage of wing pads (Figure 3O,P; Figure S7D,E). These tissue‐specific proteome alterations were further corroborated by protein staining after sodium dodecyl sulfate‐polyacrylamide gel electrophoresis (SDS‐PAGE) and LC‐MS/MS proteomic analysis (Figure S7F–K). The composite data document that the hormone‐activated NFYB orchestrates tissue allometry by promoting protein biosynthesis, likely through activating the core translational machinery that includes ribosomes and tRNAs.

### 2.4 NFYB Enhances Translational Efficiency for Protein Biosynthesis

**FIGURE 4:**
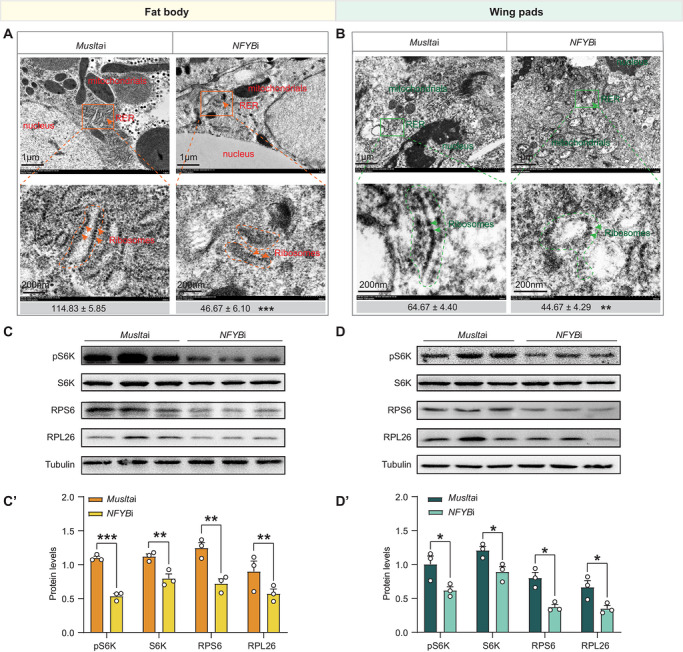
NFYB mediates protein biosynthesis through enhancing the core translational machinery. (A and B) Transmission electron microscopy (TEM) images of ER‐associated ribosomes in the fat body (A) and wing pads (B) following *NFYB* knockdown, respectively. Low‐magnification overview (upper), high‐magnification detail (lower). (C) Western blot analysis revealed reduced expression of S6K, pS6K, RPS6, and RPL26 following *NFYB* knockdown in the fat body. (C’) Quantification of protein levels in (C). (D) Western blot analysis revealed reduced expression of S6K, pS6K, RPS6, and RPL26 following *NFYB* knockdown in the wing pads. (D’) Quantification of protein levels in (D). Scale bars: 1 µm (A and B, upper), 200 nm (A and B, lower). ^*^ *p* < 0.05, ^**^ *p* < 0.01, ^***^ *p* < 0.001, compared to the negative controls.

To solidify the underlying mechanism by which NFYB activates the core translational machinery, we employed transmission electron microscopy (TEM) to assess ribosome ultrastructure in the fat body and wing pads following *NFYB* knockdown. TEM imaging revealed that *NFYB* knockdown markedly reduced the density of rough endoplasmic reticulum (RER)‐associated ribosomes in both tissues, accompanied by evident morphological abnormalities in the remaining ribosomes (Figure 4A,B). This finding provided direct ultrastructural evidence for a role of NFYB in maintaining the integrity of the core translational machinery at the cellular level. We further extended this investigation at the molecular level by characterizing the expression of key ribosomal components and regulators using western blot analysis. We focused on the ribosomal S6 kinase (S6K), which phosphorylates the 40S subunit protein RPS6 to potently stimulate translation initiation and elongation, and on RPL26, a structural component of the 60S subunit [64, 65, 66, 67]. *NFYB* knockdown significantly suppressed the protein levels of phosphorylated S6K (pS6K), S6K, RPS6, and RPL26 in both the fat body and wing pads (Figure 4C,D′). The cellular and molecular evidence show that NFYB promotes protein biosynthesis by activating the core translational machinery.

## 3 Discussion

**FIGURE 5:**
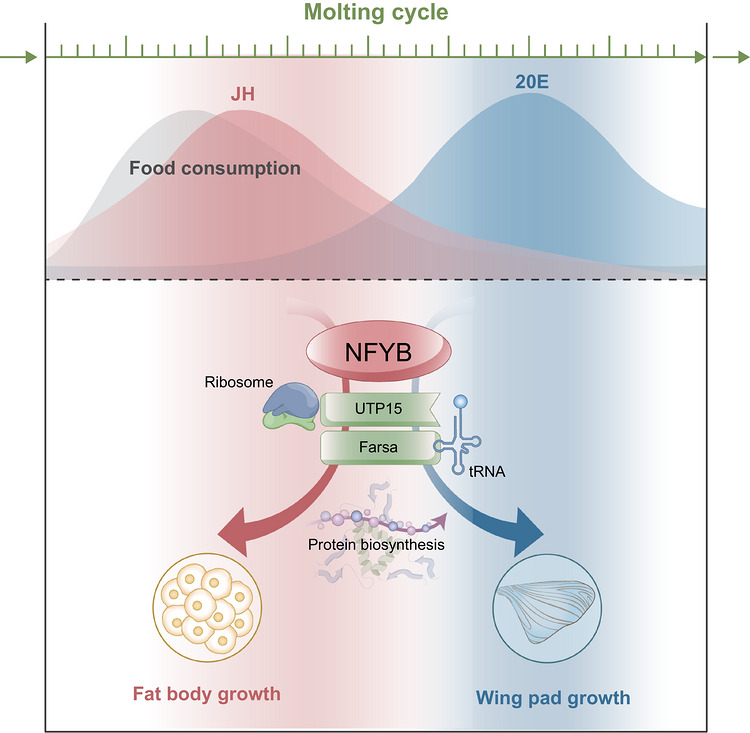
A proposed model depicting NFYB as a spatiotemporin that integrates distinct hormonal signals into distinct tissue allometry by promoting protein biosynthesis. In the fat body, JH coordinates with nutrient intake to activate NFYB during early‐to‐mid stages of the molting cycles. Conversely, in the wing pads, 20E stimulates NFYB expression during mid‐to‐late stages. This tissue‐specific, temporally distinct activation of NFYB induces the expression of ribosome biogenesis genes (e.g., *UTP15*) and tRNA aminoacylation genes (e.g., *FARSA*), promoting tissue‐specific protein biosynthesis and thereby regulating differential growth patterns.

In this study, we discovered the close correlation between body growth and feeding patterns in the American cockroach during the final five nymphal molting cycles, and further revealed that periodic changes of JH and 20E regulate the allometric growth of fat body and wing pads, respectively (Figure 1). Importantly, the transcription factor NFYB was discovered as the spatiotemporin that orchestrates distinct tissue allometry in response to periodic pulses of JH and 20E during the molting cycles (Figure 2). Mechanistically, NFYB orchestrates tissue growth by promoting protein biosynthesis (Figure 3) through activating the core translational machinery (Figure 4). These findings defined NFYB as a spatiotemporin that integrates distinct hormonal signals (JH in early fat body; 20E in late wing pads) and converges on the activation of the core translational machinery, thereby translating periodic hormonal pulses into tissue‐specific allometric growth (Figure 5).

The identification of NFYB as a spatiotemporin rests on several lines of evidence that collectively satisfy the operational criteria outlined above. First, NFYB was the uniquely common transcription factor gene emerging from an integrative transcriptomic screen encompassing stage‐specific and hormone‐induced gene sets from both fat body and wing pads (Figure 2I). This finding establishes that NFYB expression is temporally restricted to early fat body (JH‐high phase) and late wing pads (20E‐high phase) and is induced by these respective hormones (Figure 2L–O). Second, *NFYB* knockdown disrupts a convergent cellular process—protein biosynthesis—in both tissues, as evidenced by reduced de novo protein synthesis, diminished ribosomal density, and downregulation of core translational components (Figures 3I–L and Figure 4). Third, the proteomics of *NFYB* knockdown are tissue‐specific: knockdown in the fat body preferentially suppresses hexamerin storage proteins, whereas knockdown in wing pads reduces MCM complex subunits (Figure 3O,P). This indicates that NFYB not only integrates hormonal timing but also interprets the spatial context of each target tissue to direct distinct allometric outcomes. Together, these criteria distinguish NFYB from a generic downstream effector and justify its designation as a spatiotemporin—a central integrator that translates periodic hormonal signals into tissue‐specific growth programs.

In most insect species, the combination of a limited number of molting cycles, short duration in each molting cycle, and/or low efficiency of RNAi knockdown makes it difficult to identify a spatiotemporin that responds to different hormonal inputs. For example, *Drosophila* larvae undergo only three larval instars within a total of 120 h [68]. In striking contrast, the American cockroach undergoes 14 nymphal molts with its final five instars spanning ∼23 days on average [48], which provides the temporal resolution crucial for precisely delineating molting cycles and quantifying tissue growth patterns. Taking advantage of the final five nymphal molting cycles in this species, we not only characterized positive tissue allometry of early fat body and late wing pads, but also found distinct JH and 20E pulses conducted the respectively stimulatory roles. Thereafter, we performed an integrative transcriptomic analysis that included: (1) fat body across early and late stages of the final five instars; (2) wing pads across the same stages; (3) JHM‐treated fat body at the last instar; (4) 20E‐treated wing pads at the last instar. By conducting Venn analyses of these datasets, we were then able to identify NFYB as the uniquely common TF gene. This extensive screening in the American cockroach provides a powerful strategy for identifying the spatiotemporin NFYB, which links distinct hormonal cues with tissue‐specific allometric outcomes during animal ontogeny.

This screening strategy could provide a crucial reference for elucidating hormonal regulation of allometry within or across species. For example, in social insects such as termites, JH promotes allometric growth of worker head structures, including mandibles and nasus, leading to their differentiation into soldiers [36, 69, 70, 71]. Similarly, in ants, JH induces allometric growth of multiple tissues, including wing buds, ocelli, antennal and genital imaginal discs, and flight muscles in workers, facilitating their transition to a gyne (virgin‐queen) phenotype [72]. However, how JH precisely coordinates this tissue‐specific allometric growth during caste differentiation remains unclear. Using a modified strategy across multiple castes at different developmental timings will help identify such a spatiotemporin in controlling soldier differentiation. The methodology established in this study can be applied to compare tissue allometry after synchronizing the developmental stages across different species [6], as shown in our recent work in deciphering nutritional specialization and social evolution in woodroaches and termites [73]. By drawing on the methodological framework developed here, it will be possible to further identify more spatiotemporins that can help reveal the general principles governing tissue allometry within or across species.

Recent work has shown that NF‐Y preferentially occupies bookmarked genomic regions to promote transcriptional reactivation after mitosis [74] and plays essential roles in mitochondrial oxidative phosphorylation, cholesterol metabolism, and cardiomyocyte development [60, 75, 76, 77, 78, 79, 80]. Intriguingly, our study revealed a previously unrecognized function of NFYB in promoting protein biosynthesis by activating the core translational machinery. *NFYB* knockdown led to marked reductions in two hexameric complexes‐hexamerins in early fat body and the MCM complex in the late wing pads‐both of which comprise six subunits [81, 82, 83]. These results indicate that NFYB orchestrates tissue allometry by commonly promoting protein biosynthesis. Nevertheless, NFYB also exhibits tissue‐specific growth regulation. For instance, 12 h after *NFYB* knockdown, the fat body showed significant enrichment of lipid metabolism and mitochondrial‐related signaling pathways (Figure S8), which is consistent with previously reported in *Caenorhabditis elegans* [60]. In contrast, *NFYB* knockdown in the wing pads led to significant enrichment of DNA replication‐related pathways (Figure 3F), likely reflecting the central functions of MCM complexes in DNA replication, maintenance of DNA structure, and genome stability [84, 85, 86, 87].

The evolutionarily conserved NF‐Y complex, which binds the CCAAT box, is composed of three subunits, NFYA, NFYB, and NFYC [59, 88, 89]. Canonically, the NF‐Y complex is formed through the sequential assembly of its subunits: the histone‐fold containing NFYB and NFYC first form a stable heterodimer, which then recruits the DNA‐binding subunit NFYA to generate the functional trimeric complex [59, 88, 89]. However, NFYA can be replaced by several structurally homologous proteins [90, 91]. Our results suggest that NFYB not only acts alone, but also operates through the canonical NF‐Y, as all three subunits show coordinated expression changes across developmental stages and hormonal treatments (Figure S9).

In summary, our findings not only demonstrate NFYB as a spatiotemporin that integrates different hormonal signals into distinct tissue allometry in the American cockroach, but also provide a crucial reference for identifying more spatiotemporins within or across species. This study helps reveal the general principles of how appropriate body shape is achieved by tissue allometry in animals.

## 4 Experimental Section

### 4.1 Animals

The American cockroach, *Periplaneta americana*, a representative species of the order Blattodea, has been established as a model organism in entomological research [52]. Experimental specimens were reared and maintained under standardized laboratory conditions as previously described [53, 92]. Newly molted (0 – 24 h post‐molting), synchronized cockroaches were selected for experimentation, with all surgical manipulations performed under CO_2_ anesthesia to minimize procedural distress. Dissected tissues were immediately immersed in ice‐cold cockroach saline [49] to maintain cellular integrity. All experimental procedures were conducted with a minimum of three independent biological replicates.

### 4.2 Food Intake Experiment

Newly molted cockroaches from each of the last five nymphal instars (10th to 14th) were separately collected and housed in individual rearing chambers, fed with commercial rat food ad libitum. According to our established protocol [48], each instar was uniformly divided into five developmental stages (early stage 1, E1, 3 – 6 days post‐emergence; early stage 2, E2, 6 – 10 days; mid‐stage, M, 10 – 15 days; late stage 1, L1, 15 – 20 days; late stage 2, L2, 20 – 30 days) for daily measurements of food consumption and body mass. Longitudinal correlations between cumulative food intake and body weight were analyzed using Pearson's coefficient (α = 0.05). The experimental design incorporated three independent biological replicates.

### 4.3 Cell Staining and Confocal Microscopy

To observe the growth progression, fat body and wing pad tissues were collected at three defined nymphal phases per instar: Early (E, 3 – 6 days post‐emergence), Mid (M, 10 – 15 days), and Late (L, 20 – 30 days). The specific number of days was determined by the duration of the instar. Dissected tissues underwent sequential processing: (1) fixation and permeabilization in 4% paraformaldehyde with 0.6% Triton X‐100 (v/v) for 60 min at room temperature, followed by (2) three 15‐min washes in PBS‐T (0.1% Triton X‐100 in phosphate‐buffered saline). Cytoskeleton and nuclei were visualized through dual‐labeling: F‐actin networks were stained with TRITC‐conjugated phalloidin (1:400, 40734ES75, Yeasen; λex = 545 nm), while nuclei were counterstained with DAPI (1:400, 40728ES03, Yeasen; λex = 350 nm) [53]. Protein biosynthesis was assessed using the Click‐IT HPG Alexa Fluor 488 Protein Synthesis Kit (C10428, Thermo Fisher Scientific) following manufacturer protocols. Confocal imaging was performed using an Olympus FluoView FV3000 system equipped with a 40X oil‐immersion objective. Image processing utilized FV31S‐SW software (Olympus). The images were analyzed using ImageJ software. The relative growth rate (RGR) of fat body cells was calculated as: RGR = [ln(W2) – ln(W1)] / (t2 – t1), where W1 and W2 represent fat body cell area at the first (t1) and second (t2) sampling time points, respectively.

### 4.4 Tissue Weighing and Imaging

To assess wing pad growth, wing pads corresponding to the fat body dissection time points described above were collected and weighed. For wing pads at early developmental stages where weight could not be accurately determined, area was used as the primary metric. Both forewings and hindwings were measured separately and averaged for each individual, as no differential responses were observed across treatments. Tissues were imaged using a Nikon DS‐Ri2 digital camera attached to a Nikon SMZ25 stereomicroscope. Wing pad areas were analyzed and quantified with NIS‐Elements BR 4.50.00 software (Nikon). The relative growth rate (RGR) of wing pads was calculated using the formula: RGR = [ln(W2) – ln(W1)] / (t2 – t1), where W1 and W2 represent initial and final wing pad weight or area at time points t1 and t2, respectively.

### 4.5 Hemolymph Collection and Preparation

Hemolymph samples were collected directly into salinized 1.8 mL vials pre‐chilled on ice. For each sample, 100 µL of hemolymph was aliquoted, followed by the addition of 100 µL of 50% methanol and 400 µL of hexane. The mixture was vortexed for 1 min and centrifuged at 2000 ×g for 5 min at 4°C. The upper organic phase was transferred to a new silanized vial and dried under a nitrogen stream. Dried samples were subsequently resuspended in 50% methanol for LC‐MS/MS analysis or stored at −20°C for later use.

### 4.6 JH and 20E Quantification

JH quantification was performed via LC‐MS/MS using an AB Sciex QTRAP 4500 MD triple quadrupole mass spectrometer interfaced with a Shimadzu Exion LC UHPLC system. Chromatographic separation was achieved on a Waters BEH C18 column (130 Å, 1.7 µm, 2.1 mm × 50 mm). The mobile phase comprised water/acetonitrile/formic acid (solvent A: 98/2/0.1%; solvent B: 2/98/0.1%), with a linear gradient from 10% to 85% B over 10 min at 0.4 mL/min flow rate [53, 92, 93]. Detection was performed in electrospray ionization positive mode, with JH quantified via multiple reaction monitoring. For 20E quantification, the extracted 20E was analyzed using a commercial ELISA kit (ML062795, Mlbio) following the manufacturer's instructions [94].

### 4.7 JH and 20E Application and Neck Ligation

Newly emerged final‐instar cockroaches were collected for experimental manipulation. A potent JH analog, methoprene (16807, Cayman Chemical), was administered at a dose of 100 µg per individual on day 2. Fat body tissues were harvested at the middle stage of the final instar (12 days post‐treatment) for subsequent analysis and the transcriptome data were harvested 6 h post‐injection. For 20E treatment in wing pad growth assays, newly emerged final‐instar nymphs were injected with 50 µg of 20E (HY‐N6979, MCE) dissolved in 10% ethanol in PBS on day 12 post‐molt. The timing of injection and subsequent tissue collection is schematically depicted in Figure S2A. Control animals received an equivalent volume of vehicle solution. Wing pads were collected at the late stage (5 days post‐treatment) for weight and area measurements. For transcriptome analysis following 20E treatment, final‐instar nymphs at the mid‐stage (12 days post‐molt) were injected with 50 µg of 20E. Wing pads were harvested 4 h post‐injection for RNA extraction and RNA‐seq library preparation. For neck ligation (NL) experiments, 18‐day‐old cockroaches underwent NL as previously described [92]. To restore 20E activity in NL‐treated individuals, 50 µg of 20E was administered via injection immediately following ligation. Control groups received injections of an equivalent volume of vehicle solution. Wing pads were collected at the late stage (approximately 10 days post‐treatment) for further analysis.

### 4.8 Tissue Incubation In Vitro

Fat bodies, along with adherent abdominal tergites and epidermal tissues, were dissected from cockroaches at the early N14 instar. Tissues were preincubated for 30 min in 1 mL of Grace's Insect Medium (11605094, Thermo Scientific) at 30°C in the dark [95]. Following preincubation, tissues were incubated for 8 h in the medium supplemented with JHM at concentrations of 6, 9, and 12 µm. Control groups received an equivalent volume of the solvent. After the final incubation, tissues were harvested for further use.

### 4.9 RNA Interference (RNAi)‐Mediated Gene Silencing

Gene‐specific fragments (400 – 600 bp) were amplified from target gene cDNA to generate templates for dsRNA synthesis. PCR primers incorporating T7 promoter sequences were used to amplify these templates (Table S1). Double‐stranded RNA (dsRNA) was synthesized using the T7 RiboMAX Express RNAi Kit (P1700, Promega) according to the manufacturer's protocol. A 193‐bp DNA fragment of *Mus musculus* lymphotoxin‐alpha (*Muslta* gene) was subcloned to generate a phylogenetically distinct non‐targeting control construct [49, 51]. dsRNA concentrations were quantified using a NanoDrop spectrophotometer (Thermo Scientific), and 6 – 12 µg of dsRNA were administered via abdominal injection to each cockroach on day 2 post‐molt. Control cohorts received equivalent doses of non‐targeting dsRNA under identical conditions. Tissues were harvested for analysis following treatment.

### 4.10 Total RNA Extraction and Quantitative Real‐Time PCR (qPCR)

Fat body and wing pad samples were collected, and rapidly frozen in liquid nitrogen to preserve RNA integrity and prevent degradation, and stored at −80°C until further processing. Total RNA was extracted using the Direct‐zol RNA MiniPrep Kit (R2072, Zymo Research) following the manufacturer's instructions. cDNA synthesis was performed with M‐MLV Reverse Transcriptase (2641A, TaKaRa) according to the manufacturer's protocol. Quantitative real‐time PCR (qPCR) was conducted using Hieff qPCR SYBR Green Master Mix (Low Rox Plus; 11202ES03, Yeasen) on the Applied Biosystems QuantStudio 6 Flex Real‐Time PCR System (4485691, Thermo Fisher). *Actin* served as the endogenous reference for qPCR normalization, and target gene expression levels were standardized to *actin* using the ΔΔCt method [50, 53]. All the primers used for qPCR are listed in Table S2.

### 4.11 RNA‐seq and Assembly

Fat body and wing pad tissues were dissected from cockroach nymphs and immediately flash‐frozen in liquid nitrogen to preserve RNA integrity. For each biological replicate, tissues from 10 cockroaches were pooled and stored at −80°C until RNA extraction. Total RNA was isolated using the Direct‐zol RNA Miniprep Kit (R2072, Zymo Research) with an integrated genomic DNA digestion step. RNA quantity (≥1 µg per sample) was used for cDNA library preparation. Sequencing libraries were generated and sequenced on the Illumina NovaSeq 6000 platform by Biomarker Technologies. After quality filtering and duplicate removal, clean reads were aligned to the *P. americana* reference genome [49] using HISAT2. Gene expression levels were quantified as fragments per kilobase of transcript per million mapped reads (FPKM). De novo transcriptome assembly of clean reads into coding sequences was performed using Trinity with default parameters [53, 96].

### 4.12 Bioinformatics Analysis

Transcriptome analysis, including differential expression analysis with the edgeR package (fold change > 1.5, *p*‐value < 0.05), along with Gene Ontology (GO) and Kyoto Encyclopedia of Genes and Genomes (KEGG) pathway enrichment analyses, was performed using the OmicShare online platform (https://www.omicshare.com/tools). Heatmaps were visualized using TBtools [97].

### 4.13 Determination of Protein Levels and SDS‐PAGE Analysis

Fat body and wing pad samples were collected, pooled in equal amounts respectively, and snap‐frozen in liquid nitrogen. Total proteins were extracted from tissues using RIPA lysis buffer (P0013B, Beyotime Biotechnology) supplemented with 1 mM phenylmethylsulfonyl fluoride (PMSF), followed by centrifugation at 12 000 g at 4°C for 30 min to clarify the lysates. Protein was separated on a 10% SDS‐polyacrylamide gel under denaturing conditions [51]. Proteins were visualized by Coomassie Brilliant Blue staining and imaged using a Tanon 5200 imaging system (Tanon). Band intensity was analyzed with ImageJ software.

### 4.14 Western Blot Analysis

Total proteins from fat body and wing pad tissues were prepared as described above. Protein aliquots (30 µg per lane) were resolved on 10% Tris‐glycine SDS‐PAGE gels and subsequently transferred to PVDF membranes (0.45 µm, ISEQ00010, Millipore). Membranes were blocked with 5% non‐fat dry milk in TBST for 2 h at room temperature prior to immunodetection [51]. Primary antibodies were applied overnight at 4°C with the following specifications: rabbit polyclonal anti‐NFYB (1:2000, C15410241, Diagenode), rabbit monoclonal anti‐β‐Tubulin (1:5000, AF1216, Beyotime), rabbit polyclonal anti‐RPS6 (1:5000, ab40820, Abcam), rabbit polyclonal anti‐RPL26 (1:5000, PA5‐95828, Thermo Fisher Scientific), rabbit polyclonal anti‐UTP15 (1:5000, PA5‐65386, Thermo Fisher Scientific), rabbit polyclonal anti‐FARSA (1:5000, PA5‐28157, Thermo Fisher Scientific), rabbit polyclonal anti‐phospho‐S6K (Thr389) (1:5000, 9209S, Cell Signaling Technology), rabbit polyclonal anti‐S6K (1:5000, a gift from Prof. Xiaolan Fan, Sichuan Agriculture University). After three 10‐min TBST washes, membranes were incubated with HRP‐conjugated secondary antibodies (Goat Anti‐Rabbit, 1:5000, A0208, Beyotime) for 2 h at room temperature. Chemiluminescent detection was performed using Immobilon Western HRP Substrate (WBKLS0500, Millipore) with signal acquisition on a Tanon 5200 imaging system. Band intensity was analyzed with ImageJ software, normalized to β‐Tubulin loading controls [51, 98].

### 4.15 Proteome and Mass Spectrometry Analysis

Tissue samples were homogenized using an MP FastPrep‐24 homogenizer (24 × 2, 6.0 m/s, 60 s, twice) in SDT buffer (4% SDS, 100 mM Tris‐HCl, pH 7.6), sonicated, and centrifuged (14 000 ×g, 40 min). Serum/plasma was centrifuged (14 000 ×g, 20 min). Protein quantification was performed using a BCA Protein Assay Kit (20201ES76, Yeasen), and 15 µg of protein per sample was separated on 4% – 20% SDS‐PAGE gels (180 V, 45 min) after boiling with 5× loading buffer. For FASP digestion, proteins were reduced with 40 mM DTT (37°C, 1.5 h), alkylated with 20 mm IAA, and digested with trypsin (1:50 wt/wt) on 10 kDa filters. Peptides were desalted using C18 Cartridges (Empore SPE) and analyzed via Orbitrap Astral mass spectrometer (Thermo Scientific) in DIA mode (380 – 980 m/z, 240 000 MS1 resolution, 25 eV collision energy). Data were processed with DIA‐NN 1.8.1 (trypsin digestion, carbamidomethylation, oxidation/acetylation modifications, FDR ≤1%) [51].

Selected protein bands were cut from SDS‐PAGE gels and subjected to in‐gel trypsin digestion. The resulting peptides were separated via reversed‐phase high‐performance liquid chromatography (HPLC) followed by mass spectrometry (LC‐MS/MS) analysis as previously described [50, 51].

### 4.16 Transmission Electron Microscopy (TEM)

Freshly dissected tissues were immediately immersed in 2.5% glutaraldehyde at 4°C overnight. After three 10‐min washes in PBS, tissues were then fixed in a 1% osmium tetroxide solution for 3 h, washed again in 1× PBS. En bloc staining was performed with 3% uranyl acetate overnight. After washing with distilled water, the tissues were dehydrated in a graded series of ethanol. Tissues were then sequentially infiltrated with 1:1 and 1:3 ratios of acetone: resin for 12 h each, and finally embedded in pure resin. Polymerization was performed at 70°C for 2 h. Ultra‐thin sections were cut using a UC7 ultramicrotome (Leica) with a diamond knife and imaged using a Talos L120C transmission electron microscope (HITACHI) [50, 99].

### 4.17 Data Analysis

Statistical analyses were performed using IBM SPSS Statistics version 19.0. Error bars in all figures represent the standard error of the mean (SEM). For comparing two independent groups, Student's t‐test was applied to assess significant differences. Pearson's correlation coefficient was calculated to evaluate correlations between variables. Multiple group comparisons were analyzed using one‐way analysis of variance (ANOVA) followed by post‐hoc Duncan's multiple range test [53]. Statistical data visualization was generated with GraphPad Prism version 8.0.2, and figure formatting was completed using Adobe Illustrator.

### 4.18 Data and Materials Availability

RNA‐seq data have been submitted to the NCBI's Sequence Read Archive (SRA) database under the accession number PRJNA1393883. Proteomics data have been submitted to the iProX database under the accession number PXD072619. All other study data are included in the manuscript and/or Supporting Information.

## Author Contributions

S.L. and F.F.L. designed research; F.F.L., S.M.Z., S.S.X., Q.L., Z.Q.Z., Y.Q.L., W.Y.L., G.Y.Z. performed research; F.F.L., S.M.Z., S.S.X., X.S.Z., S.L. analyzed data; F.F.L., S.M.Z., X.S.Z., S.N.L., S.L. wrote the paper; E.D., assisted with proofreading and revising the paper.

## Conflicts of Interest

The authors declare no competing interest.

## Supporting information




**Supporting File**: advs75767‐sup‐0001‐SuppMat.docx.

## Data Availability

The data that support the findings of this study are openly available in Sequence Read Archive (SRA) database at Https://www.ncbi.nlm.nih.gov/sra, reference number PRJNA1393883.
