# Supplementary material for: NFYB Integrates Hormonal Signals into Tissue Allometry by Promoting Protein Biosynthesis
Source: Adv Sci (Weinh). 2026 May 20:e75767. Online ahead of print. doi: 10.1002/advs.75767 (PMC13336097; doi:10.1002/advs.75767)
Supplement: Supplementary file 1 — Supporting File: advs75767‐sup‐0001‐SuppMat.docx. [file ADVS-9999-e75767-s001.docx]

**Supporting Information for**

**NFYB integrates hormonal signals into tissue allometry by promoting protein biosynthesis**

Fangfang Liu^1^, Shiming Zhu^1^, Sishi Xia^1^, Xiaoshuai Zhang^1^, Qin Li^1^, Zhanquan Zeng^1^, Yuqing Liu^1^, Wanyin Liang^1^, Guanyu Zhou^1^, Erxia Du^1^, Suning Liu^1^, Sheng Li^1^

^1^Guangdong Provincial Key Laboratory of Insect Developmental Biology and Applied Technology, Institute of Insect Science and Technology, School of Life Sciences, South China Normal University, Guangzhou, China.

Correspondence: Sheng Li (lisheng@scnu.edu.cn) and Suning Liu (liusuning@scnu.edu.cn)

Fangfang Liu, Shiming Zhu, Sishi Xia and Xiaoshuai Zhang contributed equally to this work.

**This PDF file includes:**

Figures S1 to S9

Tables S1 to S2


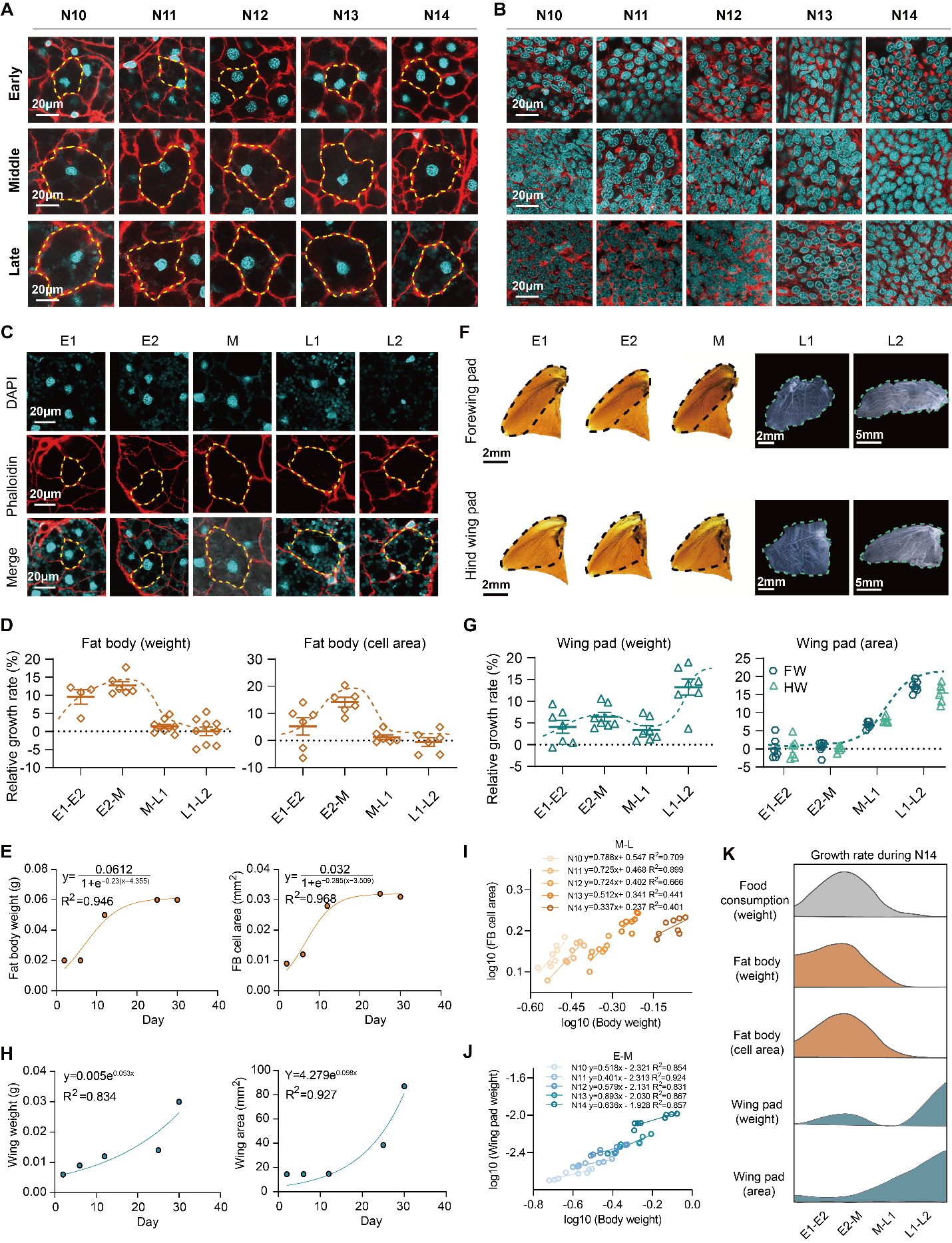


Figure S1. Developmental dynamics of fat body and wing pads across nymphal instars. (A and B) Morphologies of fat body cells (A) and wing pad cells (B) across three developmental substages (E, M, L) during the 10th to 14th instars. (C) Representative fat body cell morphology across five substages (E1, E2, M, L1, L2) in the final instar. (D) Fat body relative growth rate (weight and cell area) across five substages (E1, E2, M, L1, L2) in the final instar. (E) Sigmoidal growth model of fat body weight and cell area during the final instar. (F) Representative wing pad morphology across five substages (E1, E2, M, L1, L2) in the final instar. (G) Wing pad relative growth rate (weight and cell area) across five substages (E1, E2, M, L1, L2) in the final instar. (H) Sigmoidal growth model of wing pad weight and area in the final instar. (I) Allometric analysis between fat body and whole-body growth during mid-to-late nymphal stages. (J) Allometric analysis between wing pad and whole-body growth during early-to-mid nymphal stages. (K) Schematic of fat body and wing pad growth during the final nymphal instar. E: early stage, M: mid-stage, L: late stage. Scale bars: 20 μm (A, B, C), 2 mm (E1, E2, M and L1), 5 mm (L2).


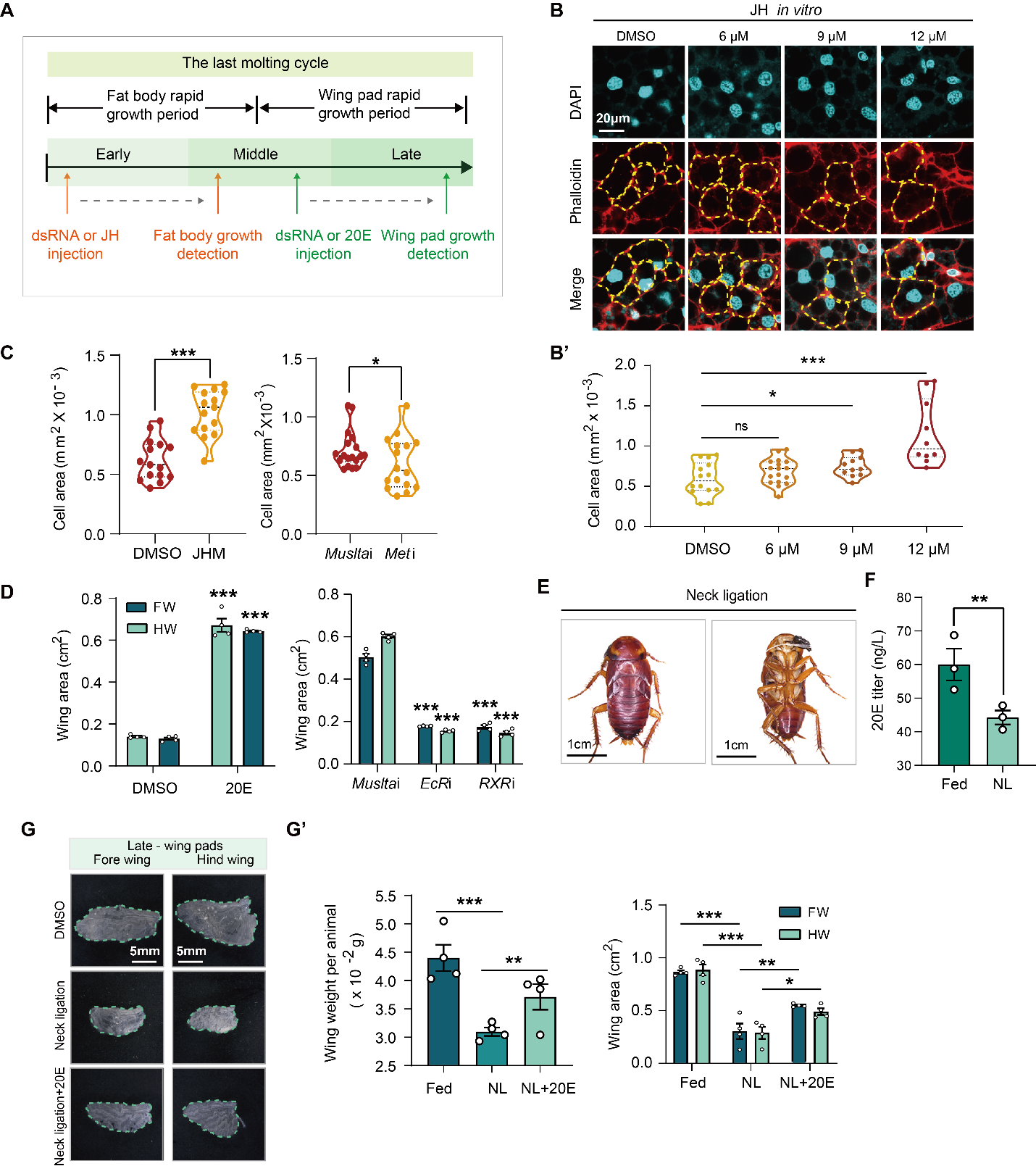


Figure S2. Differential regulation of fat body and wing pad growth by JH and 20E. (A) Experimental timeline in this study. Final-instar cockroaches were used for functional studies. To investigate the role of JH in fat body growth, *Met* dsRNA and JHM were injected at the early stage. The fat body tissues were then dissected and detected at the middle stage. To investigate the role of 20E in wing pad growth, *EcR* dsRNA, *RXR* dsRNA, and JHM were injected at the middle stage. The wing pad tissues were dissected and detected at the late stage. The following experiments were performed in accordance with these protocols in this study. (B) Dose-dependent fat body cell enlargement induced by JHM (methoprene) *in vitro*. (B’) Quantification of fat body cell area in (B). (C) Quantification of fat body cell area following JHM treatment and *Met* knockdown. (D) Wing pad area following 20E treatment and knockdown of *EcR* and *RXR*. (E) Schematic of neck ligation (NL) performed at nymphal mid-stage. (F) Reduction in 20E titer after NL quantified by ELISA. (G) Wing pad morphology after NL and subsequently restored with 20E. Scale bars: 5 mm. (G’) Quantification of wing area and weight in (G). Data are shown as mean ± SEM. FW: fore wing pad, HW: hind wing pad. Scale bars: 20 μm (B), 2 mm (D), 1 cm (F). * *P* < 0.05, ** *P* < 0.01, *** *P* < 0.001, compared to the negative controls.


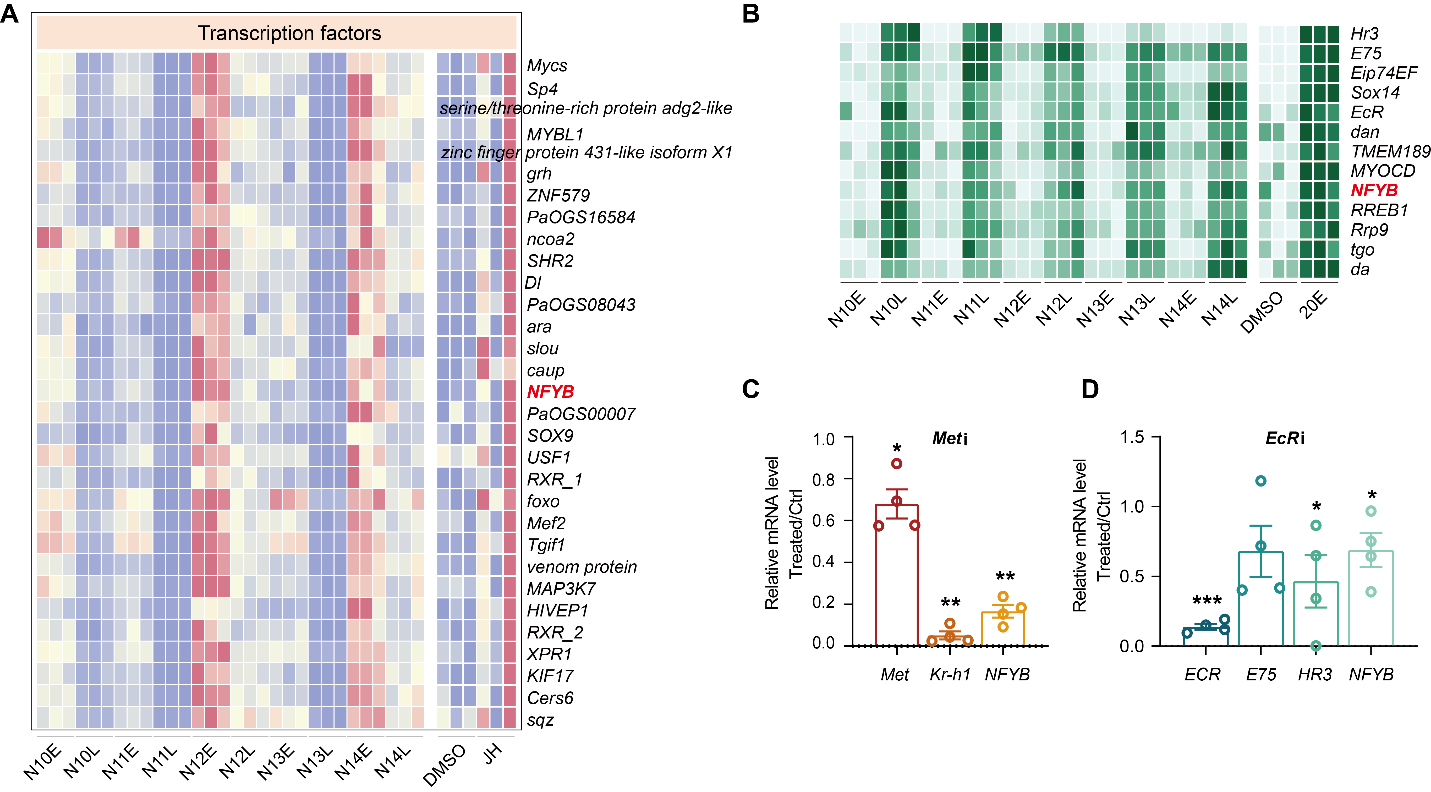


Figure S3. NFYB is a common transcription factor differentially regulated by JH and 20E signaling in fat body and wing pads. (A) Heatmap of 31 transcription factors enriched in fat body transcriptomes (from Figure 2I). (B) Heatmap of 13 transcription factors enriched in wing pad transcriptomes (from Figure 2I). (C) The expression of *Met*, *Kr-h1* and *NFYB* after *Met* RNAi in the fat body. (D) The expression of *E75*, *HR3* and *NFYB* after 20E treatment in the wing pads. Data are shown as mean ± SEM. * *P* < 0.05, ** *P* < 0.01, *** *P* < 0.001, compared to the negative controls.


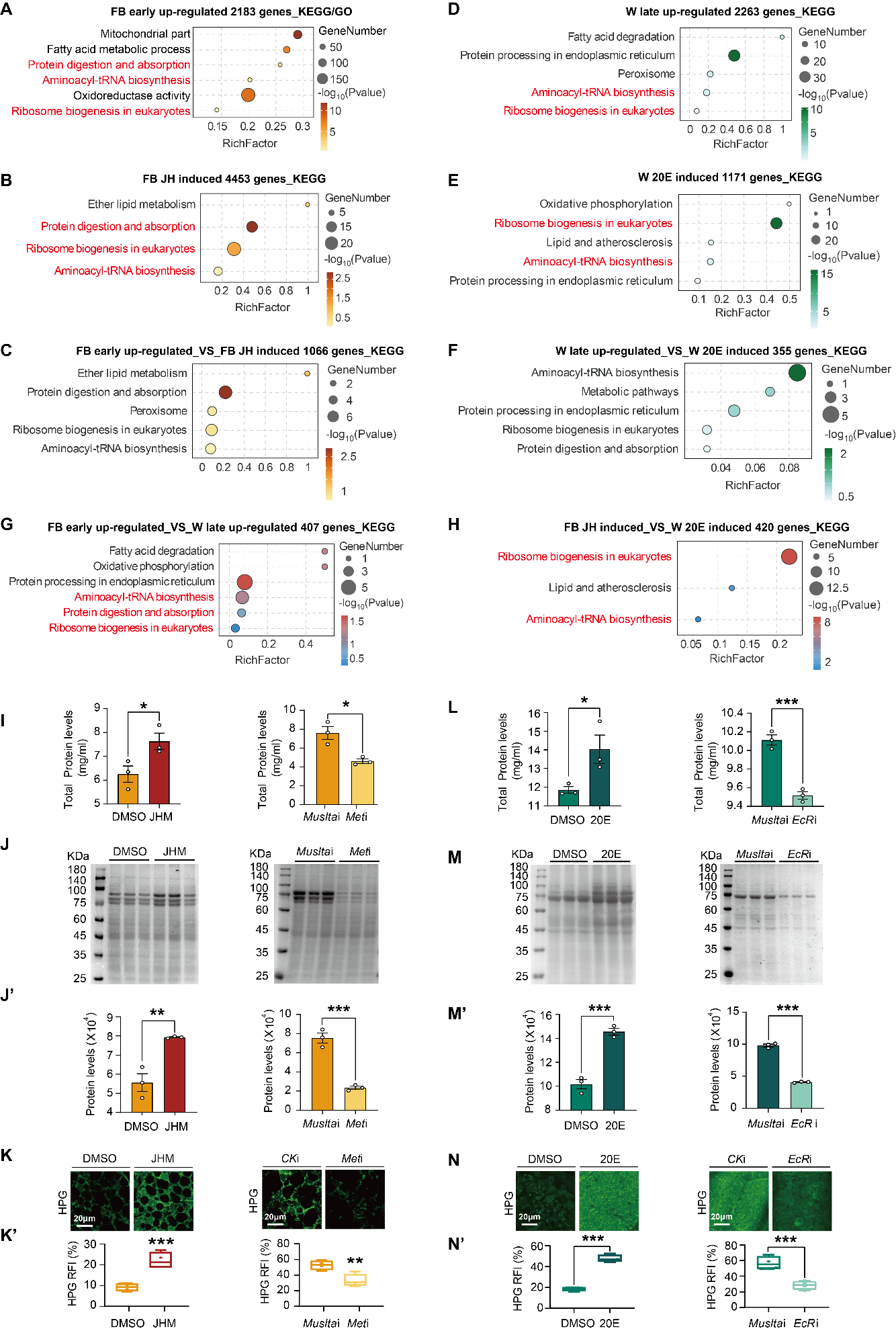


Figure S4. JH and 20E orchestrate tissue- and stage-specific transcriptomes and protein biosynthesis. (A) KEGG and GO analyses based on 2,183 upregulated DEGs in the fat body at the early stage of each instar. (B) KEGG analysis based on 4,453 JH-induced DEGs in the fat body at early stage in the final instar. (C) KEGG analysis based on 1,066 overlapped DEGs between early-instar-upregulated DEGs and JH-induced DEGs. (D) KEGG analysis based on 2,263 upregulated DEGs in wing pads at late stage of each instar. (E) KEGG analysis based on 1,171 20E-induced DEGs in wing pads at late stage in the final instar. (F) KEGG analysis based on 355 overlapped DEGs between late-instar-upregulated DEGs and 20E-induced DEGs. (G) KEGG analysis based on 407 overlapping DEGs between fat body early-instar-upregulated DEGs and wing pad late-instar-upregulated DEGs. (H) KEGG analysis based on 420 overlapping DEGs between JH-induced DEGs in the fat body and 20E-induced DEGs in wing pads. (I and J) Total protein levels in fat body after JHM treatment or *Met* RNAi, measured by BCA assay (I) and SDS-PAGE (J). (J’) Quantification of band intensity in (J). (K) Protein biosynthesis (HPG staining) in fat body following JHM treatment or *Met* RNAi. (K’) Quantification of HPG signal intensity in (K). (L and M) Total protein levels in wing pads after 20E treatment or *EcR* RNAi, measured by BCA assay (L) and SDS-PAGE (M). (M’) Quantification of band intensity in (M). (N) Protein biosynthesis (HPG staining) in wing pads following 20E treatment or *EcR* RNAi. (N’) Quantification of HPG signal intensity in (N). JHM: methoprene (JH mimic). Scale bars: 20 μm (K, N). Data are shown as mean ± SEM. * *P* < 0.05, ** *P* < 0.01, *** *P* < 0.001, compared to the negative controls.


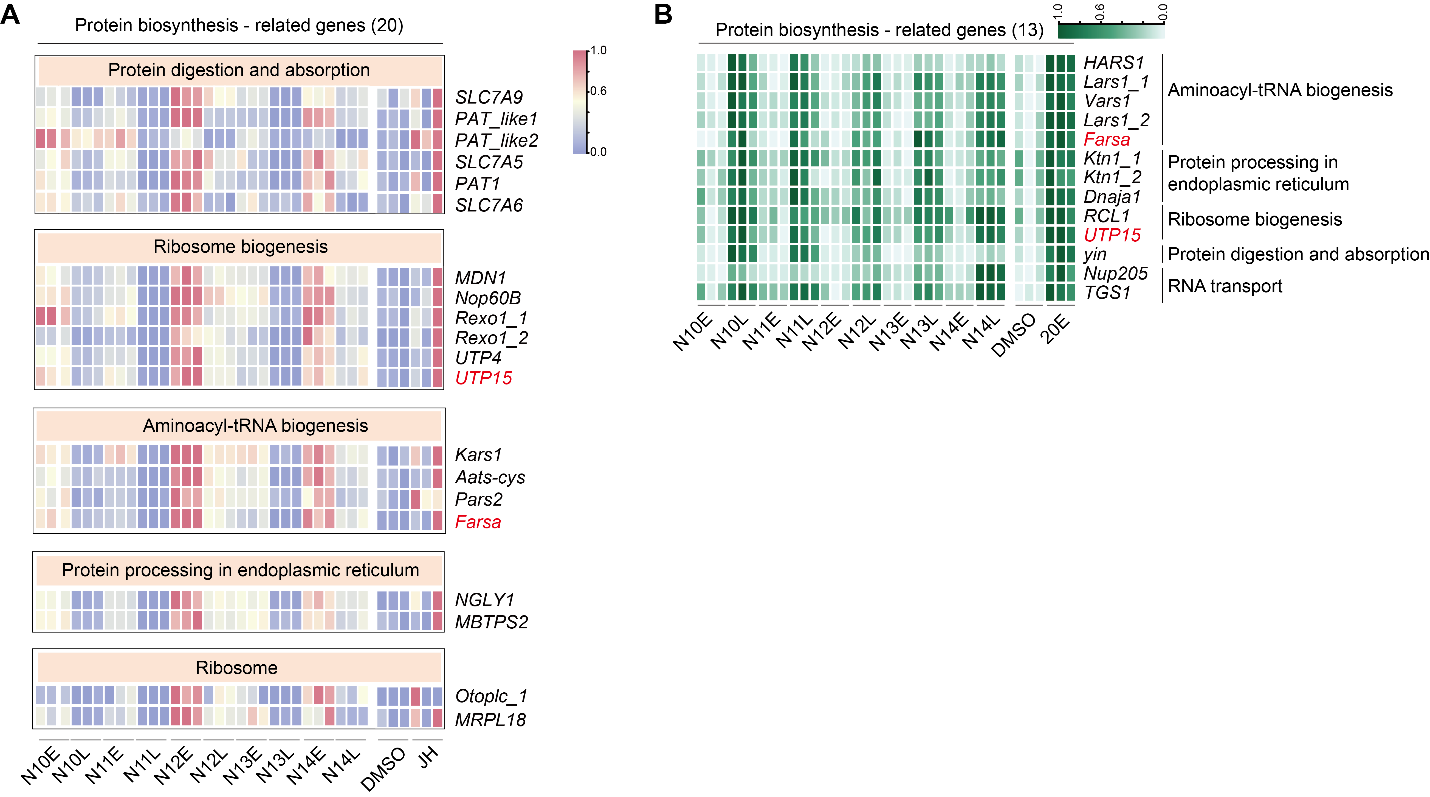


Figure S5. Heatmap of protein biosynthesis-related genes regulated by JH and 20E. (A) Heatmap of 20 protein biosynthesis-related genes enriched in fat body transcriptomes (from Figure 2I). (B) Heatmap of 13 protein biosynthesis-related genes enriched in wing pad transcriptomes (from Figure 2I).


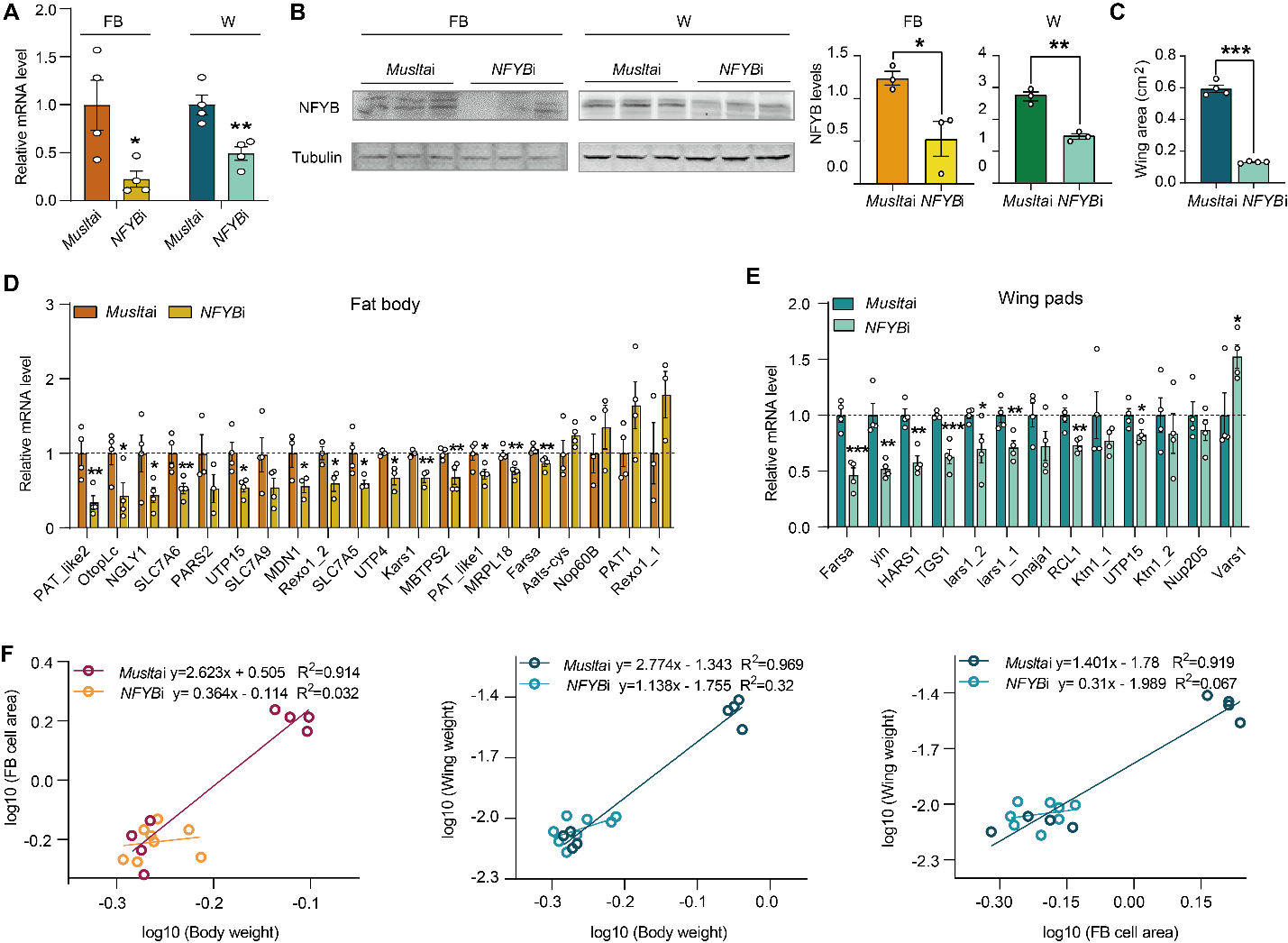


Figure S6. *NFYB* depletion represses the expression of protein biosynthesis-related genes and allometric growth. (A) Efficiency of RNAi-mediated *NFYB* knockdown in the fat body and wing pads. (B) The protein levels of NFYB in the fat body and wing pads following *NFYB* knockdown. (C) Quantification of wing area following *NFYB* knockdown. (D) Expression levels of 20 protein biosynthesis-related genes measured by qPCR in the fat body following *NFYB* knockdown (from Figure 2I). (E) Expression levels of 13 protein biosynthesis-related genes measured by qPCR in the wing pads following *NFYB* knockdown (from Figure 2I). (F) Allometric analysis of fat body versus whole-body growth (left), wing pad versus whole-body growth (middle), and fat body versus wing pad growth (right) following *NFYB* knockdown. FB: fat body, W: wing pad. Data are shown as mean ± SEM. * *P* < 0.05, ** *P* < 0.01, *** *P* < 0.001, compared to the negative controls.


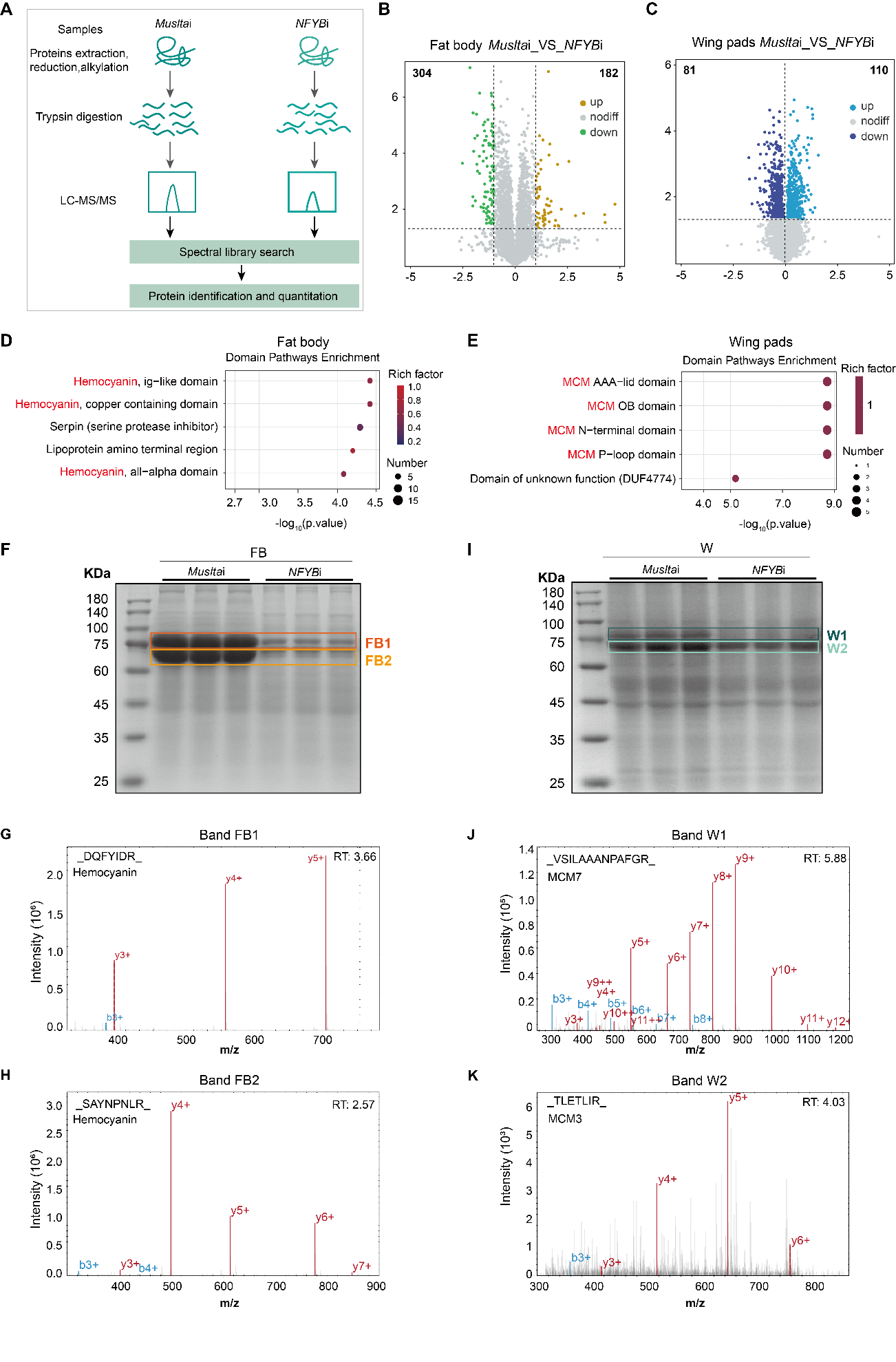


Figure S7. Proteomic analysis following *NFYB* knockdown. (A) Experimental workflow for quantitative proteomics using LC-MS/MS following *NFYB* knockdown*.* (B and C) Volcano plots of differentially abundant proteins in fat body (B) and wing pads (C) between *NFYB* knockdown and control groups. (D) Domain pathway enrichment in the fat body following *NFYB* knockdown. (E) Domain pathway enrichment in the wing pads following *NFYB* knockdown. (F) Changes of total protein levels in the fat body following *NFYB* knockdown by SDS-PAGE. (G) Mass spectrometry analysis identified fat body band 1 (FB1) as a hemocyanin in (F). (H) Mass spectrometry analysis identified fat body band 2 (FB2) as a hemocyanin in (F). (I) Changes of total protein levels in the wing pads following *NFYB* knockdown by SDS-PAGE. (J) Mass spectrometry analysis identified wing pad band 1 (W1) as MCM7 in (I). (K) Mass spectrometry analysis identified wing pad band 2 (W2) as MCM3 in (I). FB: fat body, W: wing pad.


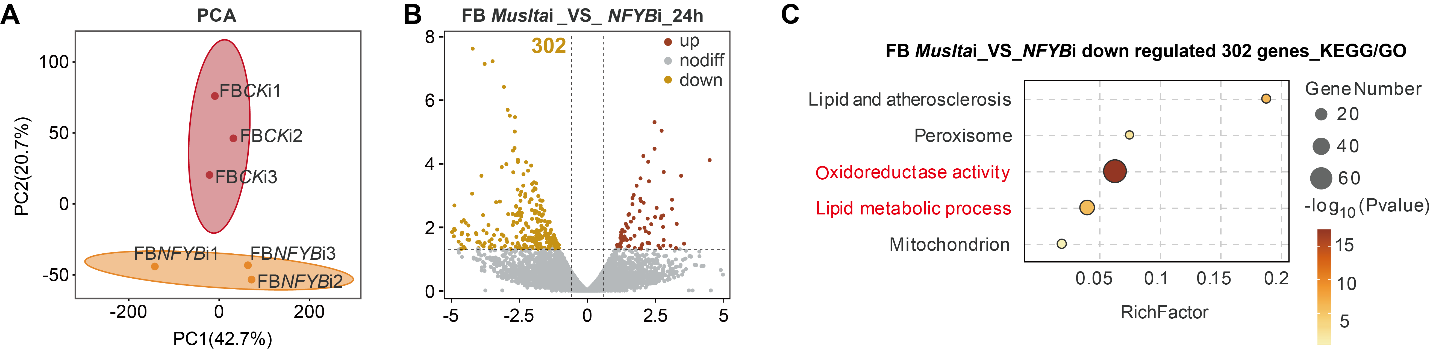


Figure S8. Transcriptomic profiling of the fat body 24 hours after *NFYB* knockdown. (A) PCA shows distinct gene expression profiles between *NFYB*-knockdown and control groups. (B) Volcano plots of DEGs in fat body between *NFYB* knockdown and control groups. (C) KEGG and GO analyses based on 302 downregulated DEGs in the fat body.


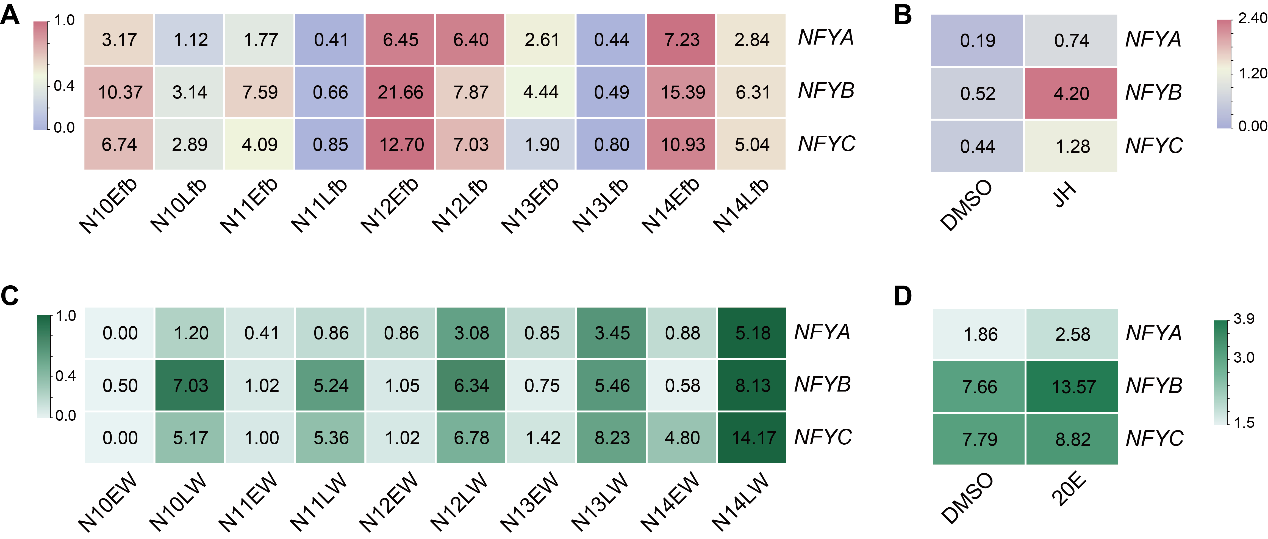


Figure S9. Expression patterns of the three NFY complexes (*NFYA*, *NFYB* and *NFYC*) and their response to JH and 20E. (A) Expression patterns of *NFYA*, *NFYB* and *NFYC* in the fat body across the final five instars. (B) The expression of *NFYA*, *NFYB* and *NFYC* in the fat body following JH treatment. (C) Expression patterns of *NFYA*, *NFYB* and *NFYC* in the wing pads across the final five instars. (D) The expression of *NFYA*, *NFYB* and *NFYC* in the wing pads following 20E treatment.

Table S1.

**Primers for PCR**

| Gene | Forward primer | Reverse primer |
| --- | --- | --- |
| *Muslta* | CACCCTCTCCACGAATTG | TAGAAGATGCTGCTGTTTCA |
| *Met* | GACTCAGATCAGCTGGAGGTAGC | CACTAATGACGTTATAACGGAC |
| *EcR* | CAAAACGAGTATGAACAACC | GAGAAAATAACAATGGCAGT |
| *NFYB* | TCACGGCAGAGAACACAGAC | ACTGTGGGATCTGGTCTGGA |

Table S2.

**Primers for qPCR**

| Gene | Forward primer | Reverse primer |
| --- | --- | --- |
| *actin* | TCCAGCTCACTGGAGAAATC | GGAGTTGTATGTGGTCTCGT |
| *NFYB* | ACAGTGAGTTACCGCCAAGT | ACCTAGCAATGGCGTGCATA |
| *Met* | CTTCCTCCCACAGGTCAGTT | GAGCTCCCGATGTGTCTGTA |
| *Kr-h1* | GCGGCCCTACAAGTGCGATAT | CGAGTTGCCCGCTCTGTATGAA |
| *EcR* | TGGACACAGCTTTGATGGCT | GAAGCTCTATCCCCGCAGAC |
| *E75* | CTGCATCTCACCGCAAACAC | ACTCGCCATCAGTGACTTGG |
| *HR3* | ACTGTTGAGAACCTGGTGGC | CGTTGTTTGGCGAGAAGGTG |
| *SLC7A9* | TCACCAGTGCTGCGAATGT | GCCAGGACAGCCGATGTA |
| *PAT_like1* | CACAGCTGGAGATGTTCCGA | TGGTTTGCCCTGAGGTTTCA |
| *PAT_like2* | TTTGTGGCTGGCAGGTGAT | AGGTCCTGCACCAGGTAGTA |
| *SLC7A5* | TGGCTAAGGTGGAAACGTCC | ATAGAAAGCGGGGAAGCCAG |
| *PAT1* | GCATTCGGCGTTCTCAACAT | ACATCTGCAGAGCGTACGAG |
| *SLC7A6* | CTGGTATGTGGGTGCTTGGT | GGCTCTGGGTTAGTGTTGCT |
| *MDN1* | CATTAGCTGTGGCTTCTGGC | CAGGGACGTCAGTACAGCA |
| *Nop60B* | GCACAGGCATTAGAGAGGCT | GCCCCAAATGCACACACATT |
| *Rexo1_1* | ACATCCGTGGCTGTTGACAT | GCCTGAGGGCTGTTCTCTTT |
| *Rexo1_2* | CATCTGCGCCTTCGAGTAGT | GCTGGTGGTAAGGCACTCTT |
| *UTP4* | CTGTGACAGCTGGTCCTTGT | GCTAGACAGAGGATTCGCCC |
| *UTP15* | ACTTGTTCTGTGCGAGTCCA | CGCCCACGCAGATTAAGTTC |
| *Kars1_1* | CTTGCCAAGTGGCATCGC | CACCAGCTGCCTTGTCCTTA |
| *Aats-cys* | TACGTGACGACATTCTGCCA | TTCACAGCCGATTTAGCACCT |
| *PARS2* | GGGAGAGGCTGAATTGCTGA | TCCTGCGTGGAGTTGAGTTC |
| *FARSA* | AATCAAAACCGCGAACGCAA | TCGTCCGACAAATGACTGCT |
| *NGLY1* | CTGAAAGCATTTGCACAACATCG | CCAGCAAGTCAATGGCATCTT |
| *MBTPS2* | TTTGGACATGCTGTTGCTGC | GCTGCCTCTTTGGATGGAGA |
| *OtopLc* | GCTAGACGCCTGGAAGAGAC | GCGGTTGTTTTTGAGCCTGT |
| *MRPL18* | TAGATCGCAGTACACGCCAC | GCCCACTCTTTCGTTGATGC |
| *HARS1* | AGCATTGGTGTAGAGCGTGT | AGTTTTGTGTCAGTACCTTAATGCC |
| *Iars1_1* | ACTTGACCATCCCATCACGG | GTCTGATCAATGCCTTCGGC |
| *Vars1* | TGTGATTGCAGTAGTTTGTGCAG | GACTGAGCATCCACAAGTCCA |
| *Iars1_2* | GGTACACTGTGTTCTCGCCA | TCGTTTCTGGACGGAGTGTG |
| *Ktn1_1* | ACACTGACAAAGGCCGTCA | CAGGTGTGTGTGAGACGTGA |
| *Ktn1_2* | TTTCGATTGTCAAGGGTGCG | ACCACGACGTCTTCCAAGTT |
| *Dnaja1* | ACACCGGCTTCACAGACAAT | AGTTGGCAGAGATGGGGTTG |
| *RCL1* | TGCAGGTTCAGGGGAAACTC | GGCCCATCAGACACTTGGAA |
| *yin* | AGGTCATCAACCCACTGCTG | CGTAAGTGTCGTAAAGGCGTG |
| *Nup205* | CATGCAGCTCAGCCATTACC | CCGCTGCCCATCACAGATA |
| *TGS1* | AGTAACACCGGAGCGCATAG | GGTCAGCAACCCCATACACA |
